# Supplementary material for: Optimizing circadian drug infusion schedules towards personalized cancer chronotherapy
Source: PLoS Comput Biol. 2020 Jan 27;16(1):e1007218. doi: 10.1371/journal.pcbi.1007218 (PMC7004559; doi:10.1371/journal.pcbi.1007218)
Supplement: S1 Text — (PDF) [file pcbi.1007218.s001.pdf]

Supplementary Information

1 PK Model Equations and Parameter Estimates, Validity of the pump-to-patient model

1.1 CPT-11

The equations for irinotecan PK model are:

$$\begin{aligned}
\frac{dL_{cpt}}{dt} &= d(t) - C_{cpt_l} * L_{cpt} - Ef_l * L_{cpt} + Up_l * B_{cpt} \\
&\quad - (V_{max}cp * L_{cpt})/(Kcp + L_{cpt}))/V_l \qquad (1) \\
\frac{dL_{sn}}{dt} &= (-C_{sn_l} * L_{sn} - Ef_l * L_{sn} + Up_l * B_{sn} \\
&\quad + (V_{max}cp * L_{cpt})/(Kcp + L_{cpt}))/V_l \qquad (2) \\
\frac{dB_{cpt}}{dt} &= (Ef_l * L_{cpt} - Up_l * B_{cpt} + Ef_o * O_{cpt} - Up_o * B_{cpt} - C_{cpt_b} * B_{cpt} \\
&\quad - (V_{max}cp * B_{cpt})/(Kcp + B_{cpt}))/V_b \qquad (3) \\
\frac{dB_{sn}}{dt} &= (Ef_l * L_{sn} - Up_l * B_{sn} + Ef_o * O_{sn} - Up_o * B_{sn} - C_{sn_b} * B_{sn} \\
&\quad + (Vcp * B_{cpt})/(Kcp + B_{cpt}))/V_b \qquad (4) \\
\frac{dO_{cpt}}{dt} &= (Up_o * B_{cpt} - Ef_o * O_{cpt} - C_{cpt_o} * O_{cpt} \\
&\quad - (V_{max}cp * O_{cpt})/(Kcp + O_{cpt}))/V_o \qquad (5) \\
\frac{dO_{sn}}{dt} &= (Up_o * B_{sn} - Ef_o * O_{sn} - C_{sn_o} * O_{sn} \\
&\quad + (V_{max}cp * O_{cpt})/(Kcp + O_{cpt}))/V_o \qquad (6)
\end{aligned}$$

where L_i, B_i and O_i represent the concentration in the Liver, Blood and Organs respectively, with *cpt* representing CPT-11, and *sn* standing for SN38.

Parameters estimates for individual patients are presented in Table A and parameter mean and CV are in Table B. Model fit was assessed through Sum of Squared Residuals (SSR) (Table C) and R^2 values (Table D). To test the validity of the PDE-based pump-to-patient model, we compared the goodness of this fit with that of the PK model with drug infusion rate equal to the infusion profile programmed in to the pump. Using the PDE to account for the properties of the system largely increased the model validity (Table C, D and Figure I)

Parameter	Patient Parameter Values										
	1	2	3	4	5	6	7	8	9	10	11
C_{cpt_l}, C_{cpt_o} (ml/h)	3035	2055	6169	6169	2491	2956	3562	1740	4842	2077	2375
C_{cpt_b} (ml/h)	4630	1635	4141	5506	1236	9338	4556	3398	3821	2650	14947
C_{sn_l}, C_{sn_o} (ml/h)	52385	21086	43837	56190	12346	57787	45700	12240	45746	16288	42194
V_{max} (ml/h)	53350	33861	1.002e06	1.21e06	34610	57870	62034	26535	77230	35604	52416
Ef_l, Up_l (ml/h)	1515	705	1895	3506	1125	1603	1506	270	1460	1022	1718
Ef_o, Up_o (ml/h)	65230	61910	1.082e05	6.238e07	61710	1.142e08	1.122e05	25600	1.768e07	65590	1.655e08

Table A: Individual Parameter Estimates of irinotecan PK model

Parameter	Mean	CV
C_{cpt_l}, C_{cpt_o} (ml/h)	3406	46.12
C_{cpt_b} (ml/h)	5077	75.35
C_{sn_l}, C_{sn_o} (ml/h)	3.68906e04	47.03
V_{max} (ml/h)	5.95558e04	48.66
Ef_l, Up_l (ml/h)	1484	53.97
Ef_o, Up_o (ml/h)	3.2756333e07	170.61

Table B: Irinotecan PK model Parameter Mean and CV across patient population

Model	Patient SSR										
	1	2	3	4	5	6	7	8	9	10	11
Original profile	28.13	142.92	91.13	226.06	456.14	143.58	32.86	15.09	70.76	76.56	140.61
PDE profile	34.08	140.97	95.57	185.59	381.72	116.13	29.51	13.2	56.29	82.65	107.69
Improvement (%)	-21.1	1.3	-4.8	17.9	16.3	19.1	10.1	12.5	20.4	-7.9	23.4
Average Improvement (%)	7.9										

Table C: Sum of Square Residuals (SSR) for irinotecan PK model, with either the original delivery profile, or that simulated throught the PDE pump-to-patient model. The table also shows improvement in percentages for each patient and average improvement for all patients.

Model	Patient R^2										
	1	2	3	4	5	6	7	8	9	10	11
Original profile	0.9	0.72	0.82	0.83	0.84	0.65	0.92	0.96	0.86	0.95	0.79
PDE profile	0.88	0.72	0.81	0.86	0.86	0.72	0.93	0.97	0.89	0.95	0.84
Improvement (%)	-2.22	0.	-1.22	3.61	2.38	10.77	1.09	1.04	3.49	0.	6.33
Average Improvement (%)	2.3										

Table D: R^2 values for irinotecan model, with either the original delivery profile, or that simulated throught the PDE pump-to-patient model. The table also shows improvement in percentages for each patient and average improvement for all patients.

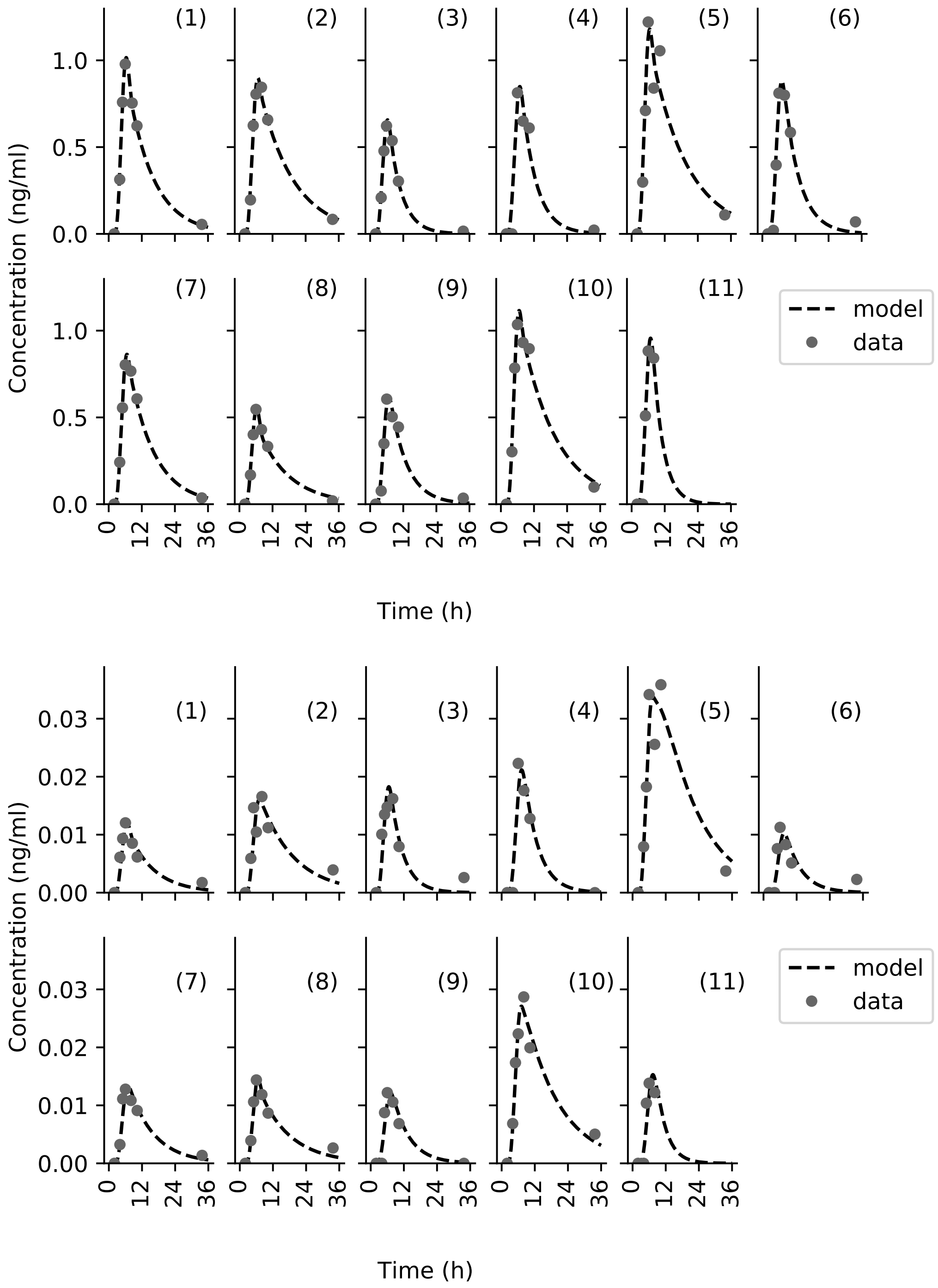

Figure A: Patient data best-fit of irinotecan PK model with original delivery profile and not PDE delivery profile. Each subplot represent an individual patient dataset, fit to the model independently. The top figure shows the fit of irinotecan plasma concentration, the bottom figure shows that of SN38, the active metabolite of irinotecan. SN38 data and model simulations include both bound and free SN38.

1.2 Oxaliplatin

The equations for oxaliplatin PK model are:

$$\frac{dL_f}{dt} = d(t) - C_l * L_f - Ef_l * L_f + Up_l * B_f - b * L_f + u * L_b)/V_l \quad (7)$$

$$\frac{dL_b}{dt} = (b * L_f - u * L_b)/V_l \quad (8)$$

$$\frac{dB_f}{dt} = (Ef_l * L_f - Up_o * B_f - Up_l * B_f - C_b * B_f - b * B_f + u * B_b)/V_b \quad (9)$$

$$\frac{dB_b}{dt} = (b * B_f - u * B_b)/V_b \quad (10)$$

$$\frac{dO_f}{dt} = (Up_o * B_f - Ef_o * O_f - C_o * O_f - b * O_f + u * O_b)/V_o \quad (11)$$

$$\frac{dO_b}{dt} = (b * O_f - u * O_b)/V_o \quad (12)$$

where L_i, B_i and O_i represent the concentration in the Liver, Blood and Organs respectively, with i representing either the the bound drug b or the free drug f.

Parameters estimates for individual patients and mean and CV over the patient population are presented in Table E. Model fit was assessed through Sum of Squared Residuals (SSR) (Table F) and R^2 values (Table G). To test the validity of the PDE-based pump-to-patient model, we compared the goodness of this fit with that of the PK model with drug infusion rate equal to the infusion profile programmed in to the pump. Using the PDE to account for the properties of the system largely increased the model validity (Table F, G and Figure II).

Parameter	Individual Patient Parameter Values											
	1	2	3	4	5	6	7	8	9	10	Mean	CV
C_l, C_o (ml/h)	1695	4.03	12.02	3.7	1.37	2.08	1.94	0.13	5159	0.21	68.80	234.41
C_b (ml/h)	5362	9.88	26.47	11.29	5.12	19.63	4.34	1.77	2.21e-04	1.10	54.42	302.50
Ef_l, Up_l (ml/h)	23939	1.15e05	1.66e06	15253	9177.12	3562	5.23e07	1373	3974	6263	5.4162e06	296.03
Ef_o, Up_o (ml/h)	18218	19071	14670	12255	10166	19192	19412	12099	114	14515	1.3971e04	41.15
b (ml/h)	9794	3934	4138	5193	5584	6090	3486	22895	4895	6273	7228	77.78
u (ml/h)	371.66	122.36	233.09	649.88	492.95	492.95	115.28	2823	921.48	475.67	670.17	115.37

Table E: Parameter Estimates of oxaliplatin PK model

Model	Patient SSR									
	1	2	3	4	5	6	7	8	9	10
Original profile	73.57	108.22	304.09	43.45	62.15	7.81	843.85	0.93	82.15	30.53
PDE profile	60.21	82.5	167.40	6.44	47.76	1.12	479.64	0.03	52.93	18.68
Improvement (%)	18.16	23.77	44.95	85.18	23.15	85.66	43.16	96.77	35.57	38.81
Average Improvement (%)	49.5									

Table F: Sum of Square Residuals (SSR) for oxaliplatin model, with original delivery profile, and oxaliplatin model with PDE simulated delivery profile. The table also shows improvement in percentages for each patient and average (mean) improvement for all patients.

Model	Patient R2									
	1	2	3	4	5	6	7	8	9	10
Original profile	0.67	0.71	0.54	0.83	0.74	0.87	0.16	0.82	0.84	0.83
PDE profile	0.66	0.67	0.83	0.97	0.73	0.98	0.53	0.83	0.87	0.84
Improvement (%)	-1.49	-5.63	53.7	16.87	-1.35	12.64	231.25	1.22	3.57	1.2
Average Improvement (%)	31.2									

Table G: R^2 values for oxaliplatin model, with original delivery profile, and oxaliplatin model with PDE simulated delivery profile. The table also shows improvement in percentages for each patient and average (mean) improvement for all patients.

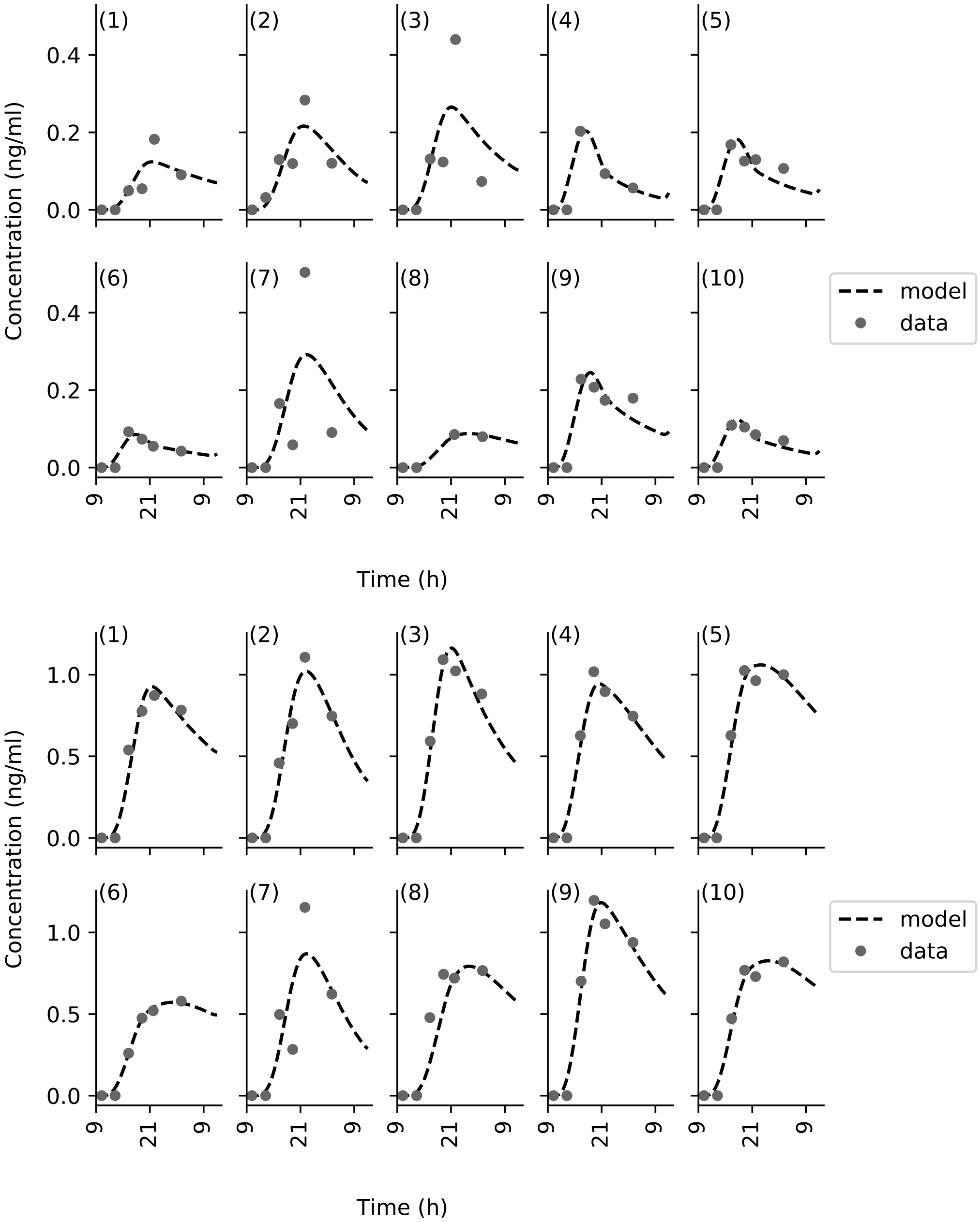

Figure B: Patient data best-fit of oxaliplatin PK model with original delivery profile and not PDE delivery profile. Each subplot is an individual patient data, fit to the model independently. The top figure shows plasma ultrafiltrate platinum concentrations, and the bottom figure shows plasma total platinum concentrations.

1.3 5-fluorouracil

The equations for 5-fluorouracil PK model are:

$$\frac{dL}{dt} = (d(t) - C_l * L - Ef_l * L + Up_l * B)/V_l \tag{13}$$

$$\frac{dB}{dt} = (Ef_l * L + Ef_o * O - Up_o * B - Up_l * B - C_b * B)/V_b \tag{14}$$

$$\frac{dO}{dt} = (Up_o * B - Ef_o * O - C_o * O)/V_o \tag{15}$$

where L_i, B_i and O_i represent the concentrations of 5-fluorouracil in the Liver, Blood and Organs respectively.

Parameters estimates for individual patients and Mean and CV over the patient population are presented in Table H. Model fit was assessed through Sum of Squared Residuals (SSR) (Table I) and R^2 values (Table J). To test the validity of the pump-to-patient model, we compared the goodness of this fit with that of the PK model with drug infusion rate equal to the infusion profile programmed in to the pump. Using the PDE to account for the properties of the system largely increased the model validity (Table I, J and Figure III).

Parameter	Patient parameter value										
	1	2	3	4	5	7	8	9	10	Mean	CV
C_l, C_o (ml/h)	132384	118360	125355	334605	349150	888311	131285	523901	117557	302323	84.18
C_b (ml/h)	84801	46116	70614	181219	86562	221789	98929	130879	36451	106373	56.0
$Ef_{o/l}, Up_{o/l}$ (ml/h)	21.09	6540	38.89	86.54	2.682e+05	1.048e+06	23.68	1.618e+06	58.18	326127	176.79

Table H: Parameter Estimates of 5-fluorouracil PK model

Model	Patient SSR								
	1	2	3	4	5	7	8	9	10
Original profile	241.2	131.1	640.4	84.4	604.3	57.6	306.8	308.2	1556.8
PDE profile	186.5	48.7	554.3	80.4	861.2	57.55	256.6	294.2	1076.4
Improvement (%)	22.6	62.9	13.4	4.7	-4.3	0.01	16.3	4.5	30.8
Average Improvement (%)	12.5								

Table I: Sum of Square Residuals (SSR) for 5-fluorouracil model, with original delivery profile, and 5-fluorouracil model with PDE simulated delivery profile. The table also shows improvement in percentages for each patient and average (mean) improvement for all patients.

Model	Patient R2								
	1	2	3	4	5	7	8	9	10
Original profile	0.79	0.96	0.73	0.85	0.69	0.86	0.75	0.64	0.83
PDE profile	0.84	0.99	0.77	0.86	0.55	0.86	0.79	0.65	0.88
Improvement (%)	6.3	3.1	5.5	1.2	-20	0	5.3	1.5	6.0
Average Improvement (%)	0.97								

Table J: R^2 values for 5-fluorouracil model, with original delivery profile, and 5-fluorouracil model with PDE simulated delivery profile. The table also shows improvement in percentages for each patient and average (mean) improvement for all patients.

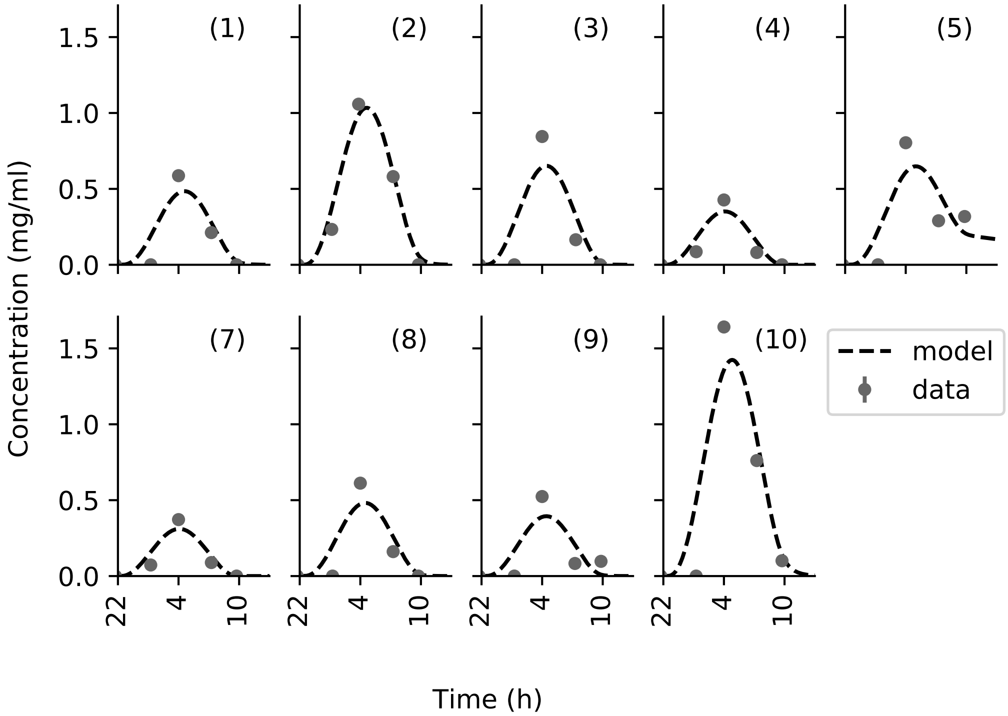

Figure C: Patient data best-fit of 5-fluorouracil PK model with original delivery profile and not PDE delivery profile. Each subplot is an individual patient data fit to the model independently.

2 Identifiability

Parameter identifiability was checked for irinotecan, oxaliplatin and 5-fluorouracil model using the method of likelihood profiles (reference [23] of main text). Briefly, the distance between the experimental data and the model is computed by an objective function, here the weighted sum of squared residuals:

$$C(\theta) = \sum_{i=1}^{m} (\frac{y_i - f(t_i, \theta)}{\sigma_i})^2$$

where y_i are the data points at the corresponding time points t_i, $f(t_i, \theta)$ are the model values at t_i with parameters θ, and σ_i the data standard deviations. Minimizing this objective function over parameter values is equivalent to maximizing the likelyhood estimator for normally distributed datasets.

For each parameter θ_j, the likelihood profile $C_{PL}(\theta_j)$ is defined as:

$$C_{PL}(\theta_j) = \min_{\theta_k \neq \theta_j} [C(\theta)]$$

The pointwise confidence interval of parameter θ_j is defined as:

$$\{\theta_j | C_{PL}(\theta_j) - C_{PL}(\theta_j^*) < \Delta_\alpha\}$$

where θ_j^* is the parameter optimal value which minimizes $C(\theta)$. Δ_α is the χ^2 distribution value for 0.95 confidence α and one degree of freedom $df = 1$:

$$\Delta_\alpha = \chi^2(\alpha, df) = 3.84.$$

A parameter is identifiable if its pointwise confidence interval is finite ([23] in main text). In other words, if the likelihood profile crosses the threshold value $C_{PL}(\theta_j^*) + \Delta_\alpha$ twice (i.e. when increasing and decreasing parameter value starting from optimal value), this proves parameter identifiability. The points at which the likelihood profile crosses the threshold are the ranges of the parameter confidence interval.

The study was done using Patient 1 parameter estimates as starting points. All model parameters were identifiable for all three drugs (Figure IV, V, VI).

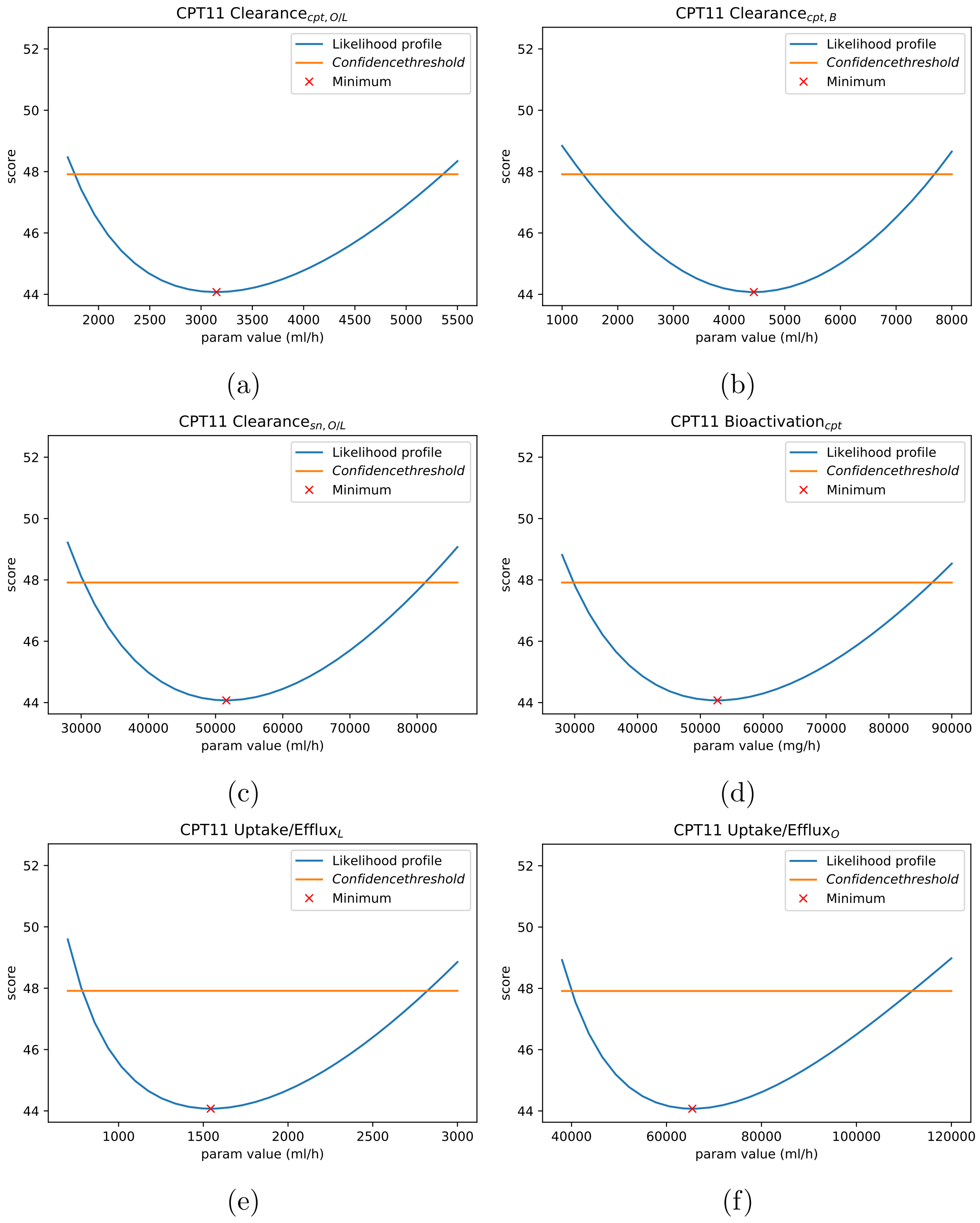

Figure D: Parameter Identifiability for irinotecan PK model.

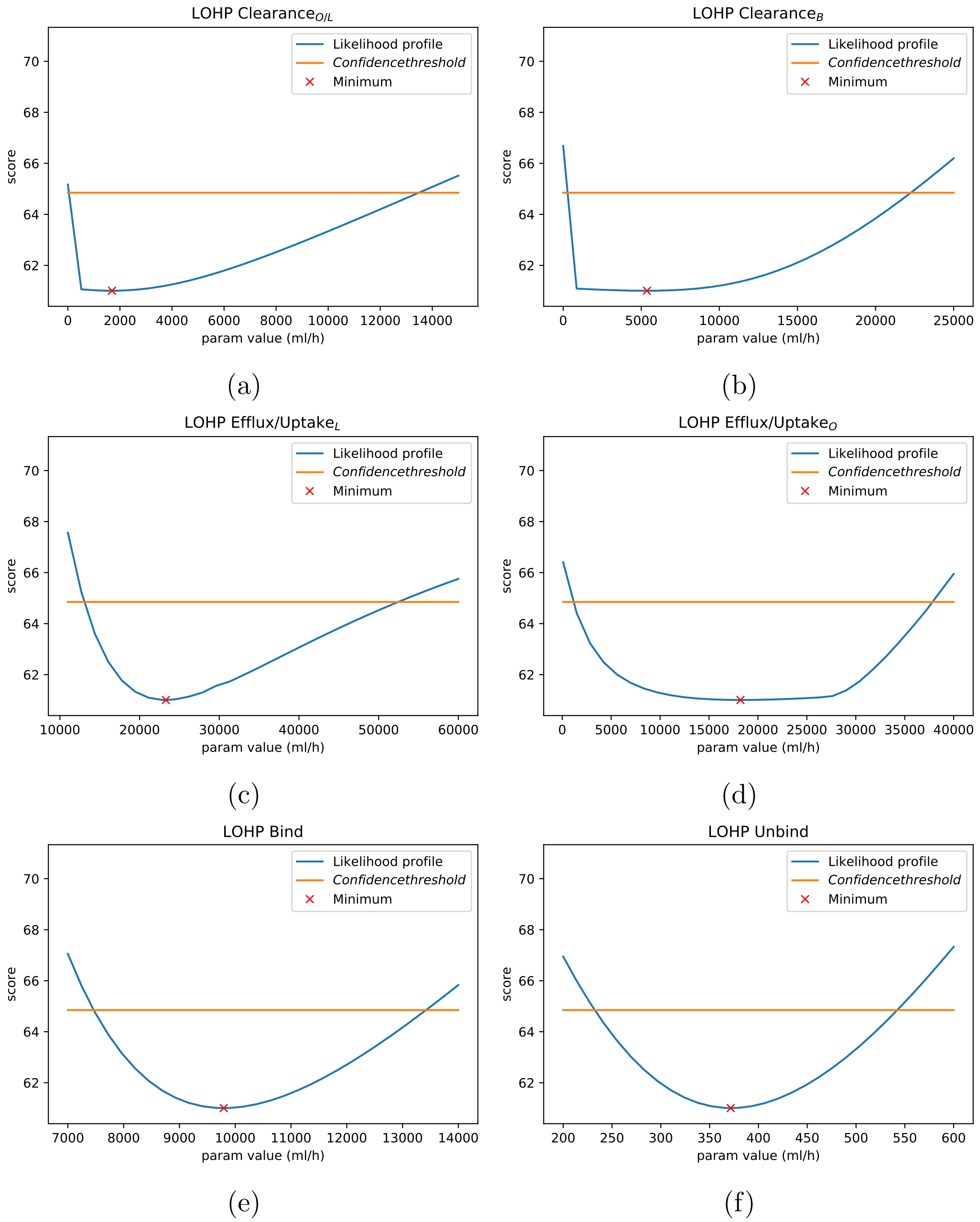

Figure E: Parameter Identifiability for oxaliplatin PK model.

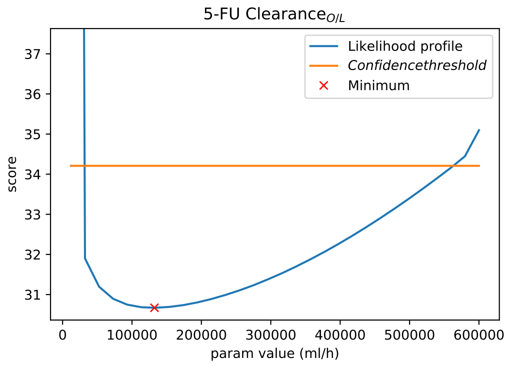

(a)

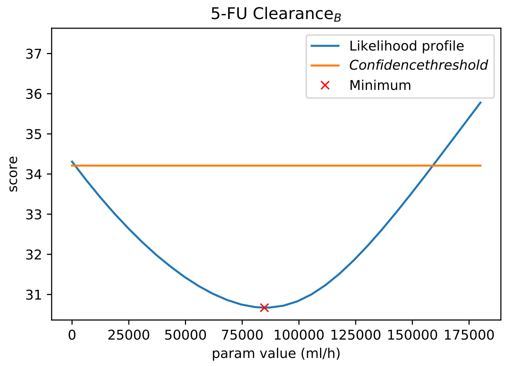

(b)

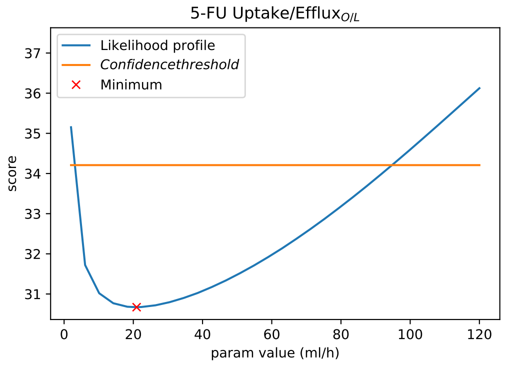

(c)

Figure F: Parameter Identifiability for 5-fluorouracil PK model.

3 Clustering

Clustering was done using fuzzy c-means clustering as described in the Methods section of main text. The validity function V_{FS} as proposed by Fukuyama and Sugeno was used to determine the number of clusters for each drug. The optimal cluster number is defined as the one minimizing V_{FS}. Results ares shown in Figures VII, VIII, IX.

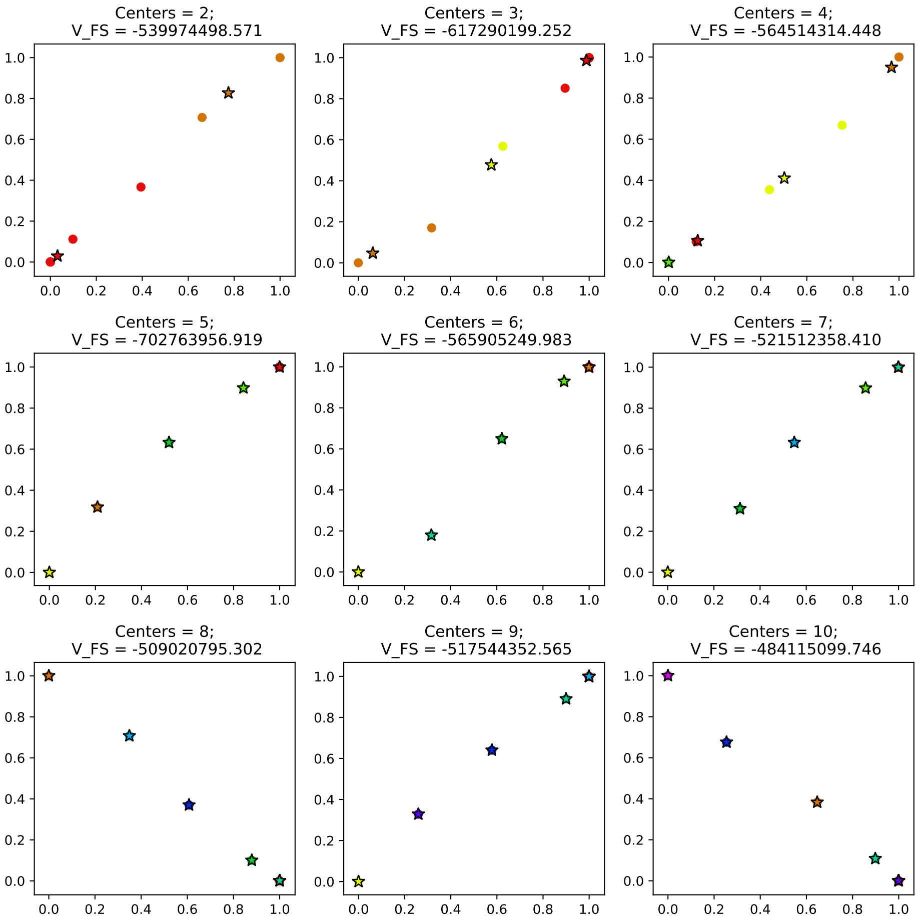

(a)

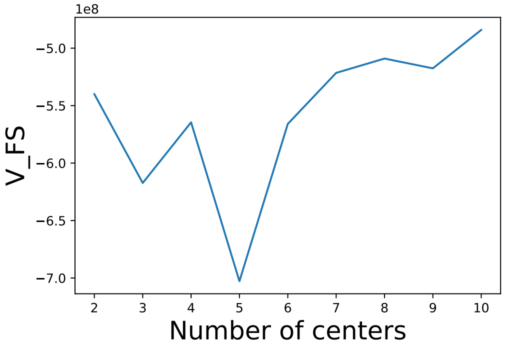

(b)

Figure G: Patient parameter clustering analysis for Irinotecan. (a) 2D vizualisation of patient clusters for different number of clusters. Centroids (stars) and patients (dots) are shown, (b) V_{FS} values for different numbers of clusters.

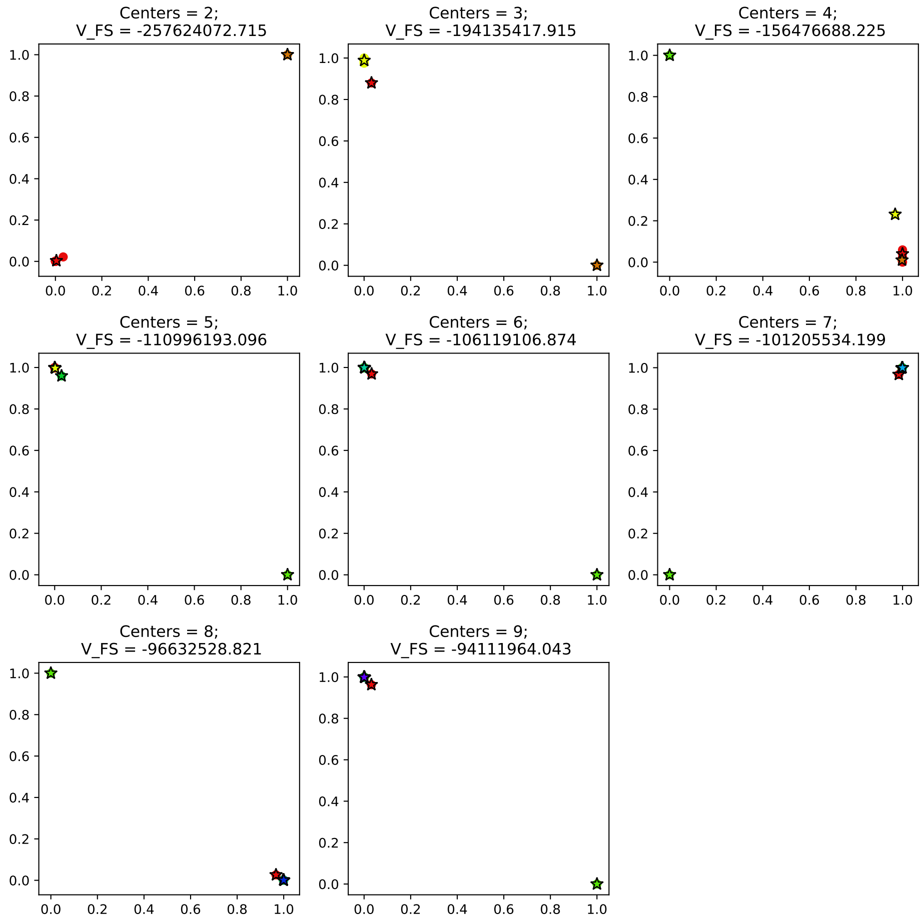

(a)

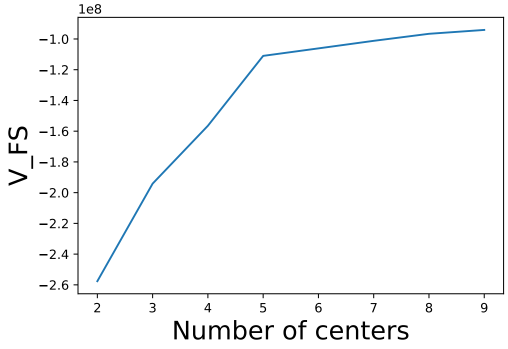

(b)

Figure H: Patient parameter clustering analysis for oxaliplatin. (a) 2D vizualisation of patient clusters for different number of clusters. Centroids (stars) and patients (dots) are shown, (b) V_{FS} values for different numbers of clusters.

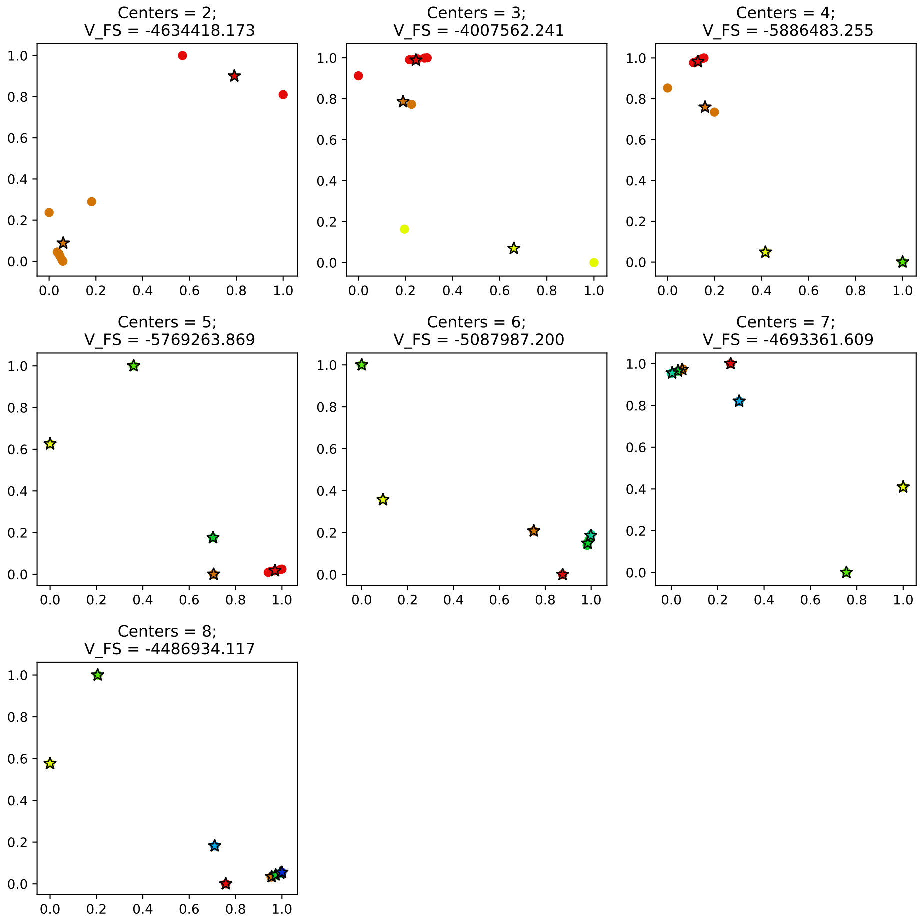

(a)

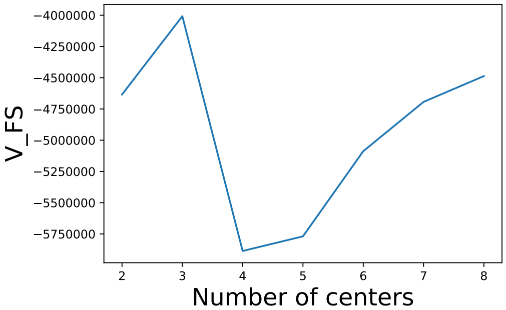

(b)

Figure I: Patient parameter clustering analysis for 5-fluorouracil. (a) 2D vizualisation of patient clusters for different number of clusters. Centroids (stars) and patients (dots) are shown, (b) V_{FS} values for different numbers of clusters.
