# Supplementary material for: Optimizing circadian drug infusion schedules towards personalized cancer chronotherapy
Source: PLoS Comput Biol. 2020 Jan 27;16(1):e1007218. doi: 10.1371/journal.pcbi.1007218 (PMC7004559; doi:10.1371/journal.pcbi.1007218)
Supplement: Attachment — Submitted filename: Response_to_reviewers.docx [file pcbi.1007218.s002.docx]

# Response to Reviewer #1’s comments:

**Major issues:
1. Wrong numbers. For examples, the mean Vmax of the irinotecan PK model (SI Table 2) should be about 2.41e05 instead of 5.96e04, and thus CV is not correct, which also affect the results shown in the main article; the mean "Cl, Co" of the oxaliplatin PK model (SI Table 5) should be 687.9 instead of 68.80; the mean Cb of the oxaliplatin PK model (SI Table 5) should be 544.2 instead of 54.42.**

Answer: We thank the reviewer for spotting these errors. I can confirm they are only typos in the transfer from Python scripts to LateX tables so that the results presented in the rest of the article are not affected. These typos have now been replaces since new simulations have been run after incorporating reviewer comments.

**2. Not appropriate statistics. For examples, when calculating CV, the authors used population standard deviation instead of sample standard deviation; using average improvement of separated SSR, CV, and R-square might not be the best choice, and the authors could try pooling the patients' data together and recompute the overall improvement, CV, and R-square.**

Answer: According to the reviewer’s comment, we have now changed *population* standard deviations to *sample* standard deviations for computing all CVs. Corrected CV values have been added to the SI and the main text. The model has now also been fit to all patient data pooled together as suggested by the reviewer and SSR and values have been added to SI along with short statement commenting on improvement in the main text. The two methods of comparing improved fit agree but the fit for the pooled data is worse hence the comparison between patient specific fit is more reliable and has been kept in the main text. line 280-281:

*“Using the pump-to-patient model allowed the improvement of the model fit to pooled data for each drug (see SI)”*

**3. Overestimated prediction performance. In Figure 6-8 (actually they are Fig 7-9 shown in my downloaded PDF file) and SI Fig 1-3, only a few data points cover the tail region. Since this article emphasize the delay effect of drug delivery in the traditional approach, more data should be collected during this period, if possible.**

Answer: Data points are available at the start of the injection and clearly validate the presence of a delay in drug administration for 5-FU and oxaliplatin (Figure 7 and 8). Regarding the tail region, data points were collected for irinotecan and validated the model prediction that the dead volume between the pump and the patient and the glucose flush had no significant effect on irinotecan PK (Figure 6). For 5-fluorouracil and oxaliplatin, we agree with Reviewer #1 that more data points covering the time of unexpected drug administration due to the glucose flush would have further validated the model which already achieved a very good fit to available data points. However, it is not surprising that the original design of the study did not include blood samples towards this tail region because they simply did not expect drug exposure and complex PK profiles during that time window. For 5-fluorouracil, unexpectedly high plasma concentrations were observed at the start of the glucose flush for patient 5 and 9 which partially validated the model. Similarly, free oxaliplatin plasma concentration displayed complex patterns with high values at the start of the glucose flush for patient 1, 2, 3 and 7 which left no doubt on the large impact of the glucose flush on oxaliplatin administration. Sentences have been added to the main text to discuss this point, line 353-358:

*“The simulations for the pump-to-patient model showed and quantified a delay between the actual start of the pump and the time when the drug appeared in the patient blood which was due to the delay needed for the drug solution to fill up the infusion tube and eventually reach the patient. A validation of this model prediction could be seen directly in the data as 5-fluorouracil and oxaliplatin plasma concentrations were close to zero for the first two times of measurements.”*

And line 376-378:

*“More data points covering the time of unexpected drug administration due to the glucose flush would have further validated the model which already achieved a very good fit to available data points. However, free oxaliplatin plasma concentration displayed complex patterns with high values at the start of the glucose flush for patient 1, 2, 3 and 7 which left no doubt on the large impact of the glucose flush on oxaliplatin administration. Similarly, unexpectedly high plasma concentrations of 5-fluorouracil were observed at the start of the glucose flush for patient 5 and 9 which partially validated the model..”*

**4. Not readable figure index. Figure 1-9 mentioned in the article are Figure 2-10 in my PDF file; Figure 10 mentioned in the article is Figure 1 my PDF file. I think the "Fig 1" in line 393 is Figure 10 mentioned in the article and is Figure 1 my PDF file. If this PDF version is different from what the authors can download from the system after they submitted, please ignore this comment.**

Answer: Figure 1 was uploaded as Figure 10 during online submission which explains the wrong figure numbers. We apologize for this and have now rectified figure indexes.

**5. Inconsistent parameters. Comparing the formula of Fukuyama and Sugeno (VFS) shown in the reference [31] and that shown in this article (at the bottom of P.14), the authors chose 1 for the parameter m, the sensitivity to the fuzzifier; however, the formula shown in the middle of P.14 assume the m = 2. This is either a typo or should be explained.**

Answer: We thank Reviewer #1 for bringing this typo to our attention. The parameter which determines sensitivity to the fuzzifier has now been properly set to 2 in agreement with the actual computations (line 575-576 page 15).

**6. In Figure 1 (Fig 2 in my PDF file), the curves of intended profile and simulated profile in sub-figure C, E, G are not shown correctly and these two curves should be swapped.**

Answer: We thank Reviewer #1 for spotting this mistake. It has now been changed so that the intended and simulated profiles have the correct colours and patterns.

**7. In line 248, it says "Blood-Liver and Blood-Organs transport parameters presented the highest CVs for all three drugs", but in SI Table 2, the CV of "Efl, Upl" is 53.97, which is not the highest two; in SI Table 5, the CV of "Efo, Upo" is 41.15, which is not the highest two.**

Answer: We agree with Reviewer #1. After the changes made to the simulations with regards to the comments from all reviewers this statement has now been altered to be (line 311-314) “In all three models the parameters which showed the largest inter-patient variability were transport parameters specifically, for irinotecan Blood-Liver transport, for oxaliplatin Blood-Organ Transport and Blood-Liver/organ transport for 5-fluorouracil.”.

**8. Figure 3-5 (Fig 4-6 in my PDF file) show three independent models, but Figure 6-8 (Fig 7-9 in my PDF file) show the concentrations of the three anticancer drugs are not 0 at many time points. Do they have interaction, either physically or statistically? Should the three models be merged?**

Answer: The administration of irinotecan, 5-fluorouracil and oxaliplatin following the reported sequence was associated to negligible drug interactions according to literature search. We have now added a sentence stating there is no significant interaction shown for the three drugs delivered in this order, line 177-179:

*“ A compartmental physiological model was designed for each drug separately, since interactions between CPT11 and LOHP, and between LOHP and 5-FU have not been demonstrated [22, 23] and CPT11 and 5-FU also showed no interaction if CPT11 is delivered first as it is in this study [24]. ”*

**Minor issues:
1. The drug rate curve of the new delivery method shown in Figure 2 (Fig 3 in my PDF file) is still a simulation result, which has not been validated in this article.**

Answer: A direct validation of the drug infusion profile predicted by the pump-to-patient model is not possible due to lack of data. However, indirect validation is provided in the form of model fit improvement when considering the pump-to-patient model. This has now been explicitly written to make sure this fact is clear to the reader, line 346-348:

*“* *Although no data were available to directly validate the model-predicted drug infusion rates, the overall framework achieved a very good fit to individual time-concentration profiles which showed model accuracy.”*

**2. Which Python package and version was used for solving PDE (line 431)? What's the version of scipy (line 435)? What's the version of sckit-fuzzy (between line 477-478)?**

Answer: The PDE were solved using a forward finite difference method which was written in python by the authors. This has now been made explicit in the text (line 511-513). *“ The PDE was solved using a backward finite difference method written by the authors within Python 3.5.2 (*[*https://www.python.org/*](https://www.python.org/)*).”* Versions of scipy and sckit-fuzzy have also been added to the text (line 515 and 567).

**3. Typo: "vhronotherapy" in line 335 should be "chronotherapy"; "sum of square residuals" in the main article and supplementary should be "sum of squared residuals"; "effux" in Fig 3-5 (Fig 4-6 in my PDF file) should be "efflux".**

Answer: These typos have now been corrected (line 406, throughout article and Figures 3-5).

**4. Format issues: "10.7\%" -> 10.7%; "tmax" in line 307; "VFS" in line 479 (FS should be subscript); "(> 0.98)" in line 484 cannot be shown correctly.**

Answer: These have all now been corrected (line 308, 478 and 582).

**5. The hyperlink shown in line 53 should be converted into an official reference format.**

Answer: This has now been converted to standard referencing (line 52).

**6. Inconsistent sub-figure index. In the article, they are in lower case; in the figures, they are upper case.**

Answer: This has now been changed (lines 115, 123, 138 and 248).

**7. The link in reference [20] doesn't work. It should be "**[**https://books.google.co.uk/books?id=5Pv4LVB_m8AC**](https://books.google.co.uk/books?id=5Pv4LVB_m8AC)**"**

Answer: This is a formatting error which has now been rectified (reference [21]).

Response to Reviewer #2’s comments

**Major Concerns:

Text:

1. Most plasma samples (4-7 time points) were collected during infusion, very few after the end of the infusion. Two post-infusion samples for CPT-11, only one for OXI and zero for 5-FU, which makes it difficult for the parameter estimates of a complex 3-6 compartmental modelling, especially the systemic drug disposition processes. For example, in the OPTILIV study (reference no.19), the plasma levels of OXI for most patients did not show any declining trend, which makes it difficult to estimate clearance-related parameters.**

Answer: The issue of being able to estimate all parameters –including the ones related to clearance- based on the available data has been addressed though model identifiability analysis. The Likelihood Profile study has shown that it is indeed possible to obtain reliable estimates for all model parameters (see SI). Drug clearance obviously occurs from the start of drug administration and as long as the compound is present in the body. It could be estimated from data although it occurred simultaneously with drug infusion. Our three models have been shown to be fully identifiable for this data and the difficulty mentioned above has been dealt with. This identifiability issue is why we limited ourselves to design semi-physiological model as compared to fully physiological model which would have not been identifiable indeed. We have added a sentence to mention this, Line 408-410:

“*The quantity of data available for this study limited the models to being semi-physiological in nature”*

**2. “Semi-mechanistic” PK modelling:
The following issues applied to the final PK models of all three drugs:
a) Equations (SI 1.1, 1.2 and 1.3) do not match their semi-physiological models (Figure 3-5); for 1.1. and 1.2. first DE, it appears that the opening parenthesis is missing, as ALL terms should be divided by V.**

Answer: We thank Reviewer #2 for bringing this to our attention. Equations have now been corrected to reflect the exact models used in the numerical computations and presented in the figures.

**b) All units for the parameter estimates listed in SI (Table 1,2, 5,8) are incorrect: For example, Ci is the equivalent of clearance from compartment I, relating change in mass/time to concentrations; therefore, the unit cannot be mg/h and it does not match Figure 10 in the main paper body either.**

Answer: We thank Reviewer #2 for pointing this out and have corrected the units in the SI tables. Those are typos and do not impact on the model design or numerical simulations.

**c) The number of parameters they estimate listed in the SI does not match their equations and models.**

Answer: There were a number of typos which have now been corrected so that the models and equations are now consistent. See response to “Minor issues” for more details.

**d) Line 159-161: They “individualized” volume in all compartment for each patient but did not explain how they did that and no fixed or estimated values reported. In the main text, “scaling” of physiological volumes is mentioned, but not elaborated further. Regardless, those were model parameters that were fixed arbitrarily, especially for the “Organ” compartment.**

Answer: Compartment volumes were not fixed arbitrarily but were estimated based on the characteristics of each patient since those parameters do correspond to actual physiological volumes. The original text stated the precise methods used which rely on integrating the patient’s characteristics (height, weight…) to estimate patient-specific organ volumes (Line 159-160). The exact equations have now been added to the SI for the sake of clarity.

**e) Line 165: The compartmental physiological model includes three compartments, Blood, Organ and Liver. Elimination clearances include renal elimination for the Blood compartment, intestinal elimination for the Organs compartment and biliary excretion for the Liver compartment; apparently no hepatic metabolism (other than SN-38 formation) is allowed. However, biliary excretion does not occur for each drug. The authors should list explicitly what the contribution of biliary excretion for each of the three drugs is assumed to be to total clearance from in-vivo ADME information. In addition, other than biliary excretion, how about liver metabolism, for many drugs, liver metabolism is non-negligible clearance. For example, CPT-11 mainly metabolized by carboxylesterase and CYP450 (ref..25, Figure 1).**

Answer: In our models, Liver clearance is defined as the combination of biliary excretion and all types of liver metabolism, except for carboxylesterases activity which is explicitly modelled in the model for irinotecan PK. We thank the reviewer for pointing out the absence of explicit definition and have now added a sentence to make this clear, line 192-197.

*“In the models, the drug clearance terms accounted for all types of drug metabolism which were not explicitly modelled (e.g. hepatic CYP450 activity) and i) renal elimination for the Blood compartment, ii) intestinal elimination for the Organs compartment and iii) biliary excretion for the Liver compartment only for irinotecan and 5-fluorouracil since it could be neglected for oxaliplatin [28-30].”*

**f) Line 165: The authors didn’t clearly explain how they differentiate elimination clearance by intestinal secretion from hepatobiliary excretion. Since the 3 drugs were administered directly into the hepatic artery, this administration route excludes the possibility of the drug metabolism in the gut before entering in the system. The only possible reason of intestinal elimination would be biliary excretion of the parent drug, and then the drug may undergo further intestinal metabolism in the gut. However, this type of intestinal elimination would be counted as biliary excretion rather than intestinal elimination. It is not clear what the intestinal elimination in the physiological model is supposed to represent.**

Answer: We thank the reviewer for bringing this ambiguity to our attention. In the models, the organs compartment does not include the intestinal lumen and only accounts for the intestinal cells composing the wall of the intestine which are exposed to the drug through blood circulation. The intestinal cells may expel the drug toward the lumen or transform the drug through metabolism, both phenomena being represented by the intestinal clearance in the models. The drug excreted through the bile directly arrive into the intestinal lumen - which is not part of the Organs compartment- and the drug recirculation was neglected. These precisions have been added to the main text (line 197-204):

*“The Organs compartment did not include the intestinal lumen and only accounted for the intestinal cells composing the wall of the intestine which were exposed to the drug through blood circulation. The intestinal cells may expel the drug toward the lumen or transform the drug through metabolism, both phenomena being represented by the intestinal clearance in the models. The drug excreted through the bile directly reached the intestinal lumen - which was not considered as part of the Organs compartment- and the drug recirculation was neglected.”*

**g) Line 171: “For the sake of simplicity, uptake and efflux rate constants were assumed to be equal for Blood-liver and Blood-Organs transport”. Any evidence or in-vivo/in-vitro data to make such assumptions? No relevant references are cited in the paper. More general, the terms “uptake” and “efflux” typically assume drug transport across biological membranes, while the in-vivo transfer across compartments also involved blood flows/organ perfusion; a more general terminology should have been used.**

Answer: We thank Reviewer #2 for highlighting this issue. The transport rate constants were assumed to be passive and did not take into account further complex interactions with membrane transporters due to lack of adequate data. This assumption was made for the sake of simplicity and identifiability. We have now reworded the main text (Line220-223):

*“All transports in between compartments were considered as passive and were represented by linear terms. Throughout this paper, the terms Blood-Liver or Blood-Organ transport represent a bidirectional transport that encompasses the transfer across the blood vessel walls into/from tissues. This simplification has been adopted due to lack of data on the transport processes between compartments.”*

Next, throughout the text, “uptake” and “efflux” were replaced by Blood-Liver or Blood-Organs transport when appropriate.

**h) Line 175: Why was organ clearance assumed to be equal to liver clearance? Ref. 24 was not properly cited here. No relevant references cited in the paper support the assumptions of organ clearances equal to liver clearances for CPT-11 and OXI.**

Answer: We thank Reviewer #2 for highlighting this misleading sentence. Organs and Liver clearance were not assumed to be equal but proportional to compartment volumes when appropriate. The overall idea is to add biological information to parameter estimation procedure to ensure model identifiability. See below for more information on the added constraints.

Regarding reference 24, the sentence has been corrected as (line 213-214):

“Organ clearance was neglected for 5-fluorouracil which is mainly cleared through hepatic metabolism, biliary excretion and renal elimination”.

**1) CPT-11
(a) For the transfer/conversion between the CPT-11 and SN-38 compartments, the authors used mass concentrations rather than molar concentrations, assuming the same molecular weight for CPT-11 and SN-38. This is incorrect!**

Answer: The conversion factor has now been added to the SI equations with a sentence explaining this. “The weight conversion factor $0.67$ in (6) and (10) is to account for the difference in molecular weight of SN38 compared to CPT11.”

**(b) The authors assumed that volumes of CPT-11 and SN-38 are the same (see equation SI 1.1 (1) and (2), applying to Blood compartment and Organ compartment, too). No evidence is provided to support these assumptions.**

Answer: Compartment volumes correspond to physiological organ volumes and are therefore assumed identical for both the parent drug CPT11 and its metabolite SN38.

**(c) CPT-11 is the prodrug for SN-38, converted by carboxylesterase (CE) in the liver. Other than Liver, the authors also assumed CPT-11 is metabolized in Blood and Organ, and they share the same Vmax and Kcp values. No literature is provided to support this assumption**

Answer: CPT11 conversion into SN38 was assumed to occur in the Liver and Organs compartments but not in the Blood as carboxylesterases are not expressed in blood cells in humans (Wang et al 2018). Literature has now been provided to support this assumption as requested. The schematic has been updated (Figure 3) and a sentence has been added (Line 251-253) :

*“Irinotecan was converted into SN38 via Michaelis-Menten kinetics within the liver and organs, but not in the Blood since the activation enzymes carboxylesterases are not expressed in blood cells in humans [32]. “*

The Vmax parameters were assumed to be different in the Liver and Organs compartments and were estimated for each patient independently in order to capture inter-patient variability in CPT11 PK. The Km was set to the same value for all patients and all compartments based on in vitro studies, see answer to comment 1(d) hereafter.

**(d) Line 197-198: the authors took the Km value (presumably Kcp used in equations, authors need to make their parameters consistent) from an in-vitro study using Caco-2 cells, not hepatocytes. On top of that, no text indicates that if and how they did in-vitro-in-vivo extrapolation from cited in-vitro study. Additionally, the plasma/blood concentrations (and possibly other compartments as well) are below the Kcp value, suggesting that the MM reaction may be reduced to a simple first-order reaction (consistent with the observed PK dose-proportionality for CPT-11 after IV clinical doses, see refs 25 and 26). Finally, by fixing vmax, Kcp (or more likely vmax/Kcp), the authors are likely ignoring a major source of intra-/interpatient PK variability in CPT-11 PK - as they cannot estimate it for each patient separately!**

Answer: We thank the reviewer for raising the issue of the Km value being evaluated in intestinal cells and we have now taken a value from an in vitro study in human liver cells [33]. Km is now equal to 52.9µM and CPT11 concentrations are in the range of 1.7 µM which is low for the Michaelis Menten kinetics however since we want the model to be physiologically realistic as possible, be able to work for larger doses of CPT11 and the change of kinetics does not reduce the number of parameters we have chosen to keep this as it is. Since Km primarily reflects the affinity of the substrate and the enzyme, it is classically considered to be unchanged from in vitro to in vivo. We have added this information and references of other studies which have taken this approach in the text (Line 253-257):

“*The parameter estimate K_m=52.9µM which reflects the affinity of the substrate and the enzyme was taken directly from an in vitro study in human liver cells [33], thus making the assumption that K_m values are unchanged from in vitro to in vivo as classically done in the literature [34, 35].”*

**(e) Line 186: “34% renal elimination pathway” supported by a mass balance study with I.V. infusion of 125 mg/m2 CPT-11 to cancer patients (ref. 25). However, 51% of the total administered dose excreted by bile is not a proper statement. From their cited reference, “Fecal excretion accounted for 63.7±6.8 (range: 54.2–74.9%, n=7) of dose, whereas urinary excretion accounted for 32.1±6.9% (range: 21.7–43.8%; n=7) of dose.” Fecal excretion does not mean 100% parent drug excreted through bile. CPT-11 is mainly metabolized by the liver to SN-38 and also by CYP450 (supported by ref. 25, Figure 1).**

Answer: We thank Reviewer #2 for spotting this inconsistency. CPT11 clearance have now been set according to ref 25 to 15% through SN38 conversion in either Liver or Organs compartments, 25% for renal elimination. The sum of intestinal and liver clearance in the model corresponded to the fecal excretion estimated to 60-70% in ref 25. We have now explained this in the text (line 227-229): “For irinotecan, CPT11 drug amount though renal clearance and though combined intestinal elimination and biliary clearance were respectively set to 25% and 60% of the total administered dose [29] ”

**(f) Line 188: The authors set the parameters to 15% of the total CPT-11 dose eliminated either through liver or organ and got the same final liver and organ clearance for SN-38 (SI Table 1.), which is a not physiologically relevant estimate (ref. 25).**

Answer: The amount of activated CPT11 was estimated as the sum of SN38 and SN38G recovered in ref 25 which was approximately equal to 15% of the total administered dose. On the opposite, we agree with Reviewer #2 that SN38 liver and organs clearance cannot be set equal. Indeed, according to ref 25, SN38 does not undergo biliary excretion so that in our model SN38 liver clearance only accounts for SN38 deactivation into SN38G. On the opposite, SN38 intestinal clearance includes both SN38 deactivation and sN38 efflux by intestinal cells towards intestinal lumen. According to Ref 25, hepatic SN38 conversion into SN38G would account for maximum 4% of the total dose, and SN38 efflux would be 9% of total dose. For these reasons, we set SN38 Organs clearance rate twice as large as SN38 Liver clearance rate. We have redone the simulations and have included this in the text ( line 229-237): “As SN38, which is the active metabolite of CPT11, renal elimination was documented as negligible, the metabolite was considered to only be cleared through Liver, via metabolism into SN38G, or Organs clearance which represents both SN38 deactivation and efflux towards intestinal lumen. these cleared amounts were assumed to account for 15% of the total administered dose of irinotecan [29]. The amount of SN38 cleared via metabolism in the liver accounted for approximately 4% of the total administered does of irinotecan whereas SN38 excretion into the intestinal lumen accounted for approximately 9\% of the total dose of irinotecan. Therefore we set the SN38 clearance via organs to be twice that of the liver clearance [29].”

**(g) SSR for CPT-11 PK model (SI, Page 3). Presumably CPT-11 and SN-38 plasma concentrations were fit simultaneously to obtain the fits shown in Fig 1. And parameter estimates in Tables 1. and 2. In scaling SSR, how did the authors incorporate the large different concentration scale between CPT-11 and SN-38?**

Answer: We thank Reviewer #2 for bringing this missing details to our attention. The weighting used has now been added to the methods section. (line 517-521):

*“For the fit of the data of a given patient, the residuals were weighted by an estimated measurement error of 10% inline with precision values of the assay methods [45-47]. This method allowed to correct the residuals to be of the same order of magnitude for the parent drug CPT11 and the metabolite SN38, or for oxaliplatin free and bound concentrations”*

**2) OXI:
(a) The semi-physiological model the authors used assumed first-order association and dissociation, i.e., reversible binding, of free and bound OXI, characterized by parameters “b” and “u”. However, literature reports that platinum binds irreversibly to plasma proteins and covalent binding to tissue (ref. 26). The authors seem to misinterpret the cited literature and did not incorporate the irreversible binding in their model.**

Answer: Oxaliplatin protein binding is indeed irreversible but protein degradation and natural cell death release a very small quantity of Platinum over time as shown both in the clinics [28] and in ex vivo blood PK studies Pendyala et al 1993. In the latter investigations, the equilibrium values for free Platinum were small yet different from zero which clearly demonstrated that there was indeed a small unbinding reactions that was occurring. This phenomenon was observed for increasing drug concentrations which ruled out the hypothesis of protein saturation by the drug. Our parameter estimation procedure confirmed that parameters for binding and unbinding were at least an order of magnitude different. A sentence has been added to the text to explain this part (line 266-267)

“Free Pt could bind to proteins and unbinding from proteins, due to protein degradation [28], which was included in all compartments (Fig 4)”

**(b) Line 190-191: “The amount of Pt bound within the Organs or within the Liver was set to 84% and 12% of the total dose, respectively.” The authors set the parameters of 84% and 12% from a study in mice (ref. 27) without discussing potential inter-species differences. Regardless, it is not clear how the authors came up with 84% and 12% from the cited paper.**

Answer: We thank the reviewer for bringing the ambiguity to our attention. We have now added an explanation on how these figures have been derived from the quoted source (Line 240-247). “The Boughattas et al paper was used to estimate tissue concentrations, no data to our knowledge exists for humans so we have used this mouse data as a best approximation. The amount of platinum in the tissues was calculated from total amount found in the respective organs relative to total dose.”

**(c) Clinical data show OXI to be equally cleared by tissue distribution and glomerular filtration from plasma (ref. 27). However, the authors assumed equally “efflux” and “uptake” to Organ. Their parameter estimates of blood clearance (Cb) and organ uptake (Upo) in SI table 5 showed very different values, which contradicts the results from the clinical study. The authors assumed equal liver and organ clearance without citing any clinical evidence in support.**

Answer: We thank Reviewer #2 for spotting this inconsistency. According to ref 27, oxaliplatin is mainly cleared by tissue binding or renal elimination. Hence, we have neglected the clearance routes though the Liver or Organs and have removed any constraint on relative percentage for clearance routes, see line 213-215 ”Organs clearance and liver clearance was neglected for oxaliplatin since majority of platinum is cleared via renal clearance and the total amount cleared after the end of treatment was set to 54\% in line with the literature [20]”

**(d) Line 205: the authors assumed the same PK properties between metabolite and OXI? Is there any evidence or relevant references to support this assumption?**

Answer: Unfortunately, there is little information about the specific PK of oxaliplatin metabolites which is why we had to make this simplifying assumption. We have now stated that in the text (line 263-266):

*“In the absences of any data on the dynamics of these different metabolites they were all assumed to have the same PK properties in the model.”*

**3) 5-FU
(a) Line 193: The authors assumed 80% of total dose cleared through (“though” in text need to be corrected to “through”) liver. According to their cited ref. 24, “5-FU is eliminated primarily by hepatic metabolism, with less than 5% of the drug excreted unchanged in the urine”; the statement from the manuscript that “5-FU was shown to be mainly cleared through hepatic metabolism” is true. However, considering their early text (Line 165), they set liver biliary excretion to 80% for 5-FU is not proper. On top of that, they assume the same liver and organ clearance (see SI table 8), which does not match clinical findings.**

Answer: The typo has been corrected (line 191). According to ref 24, no organ clearance was assumed for 5-FU but this was not properly explained in the text and SI. This has now been corrected. 5-FU liver clearance in the model accounted for both hepatic metabolism and biliary excretion which correspond to 80% of the total dose according to ref 24. We have now better define liver clearance terms (line 192-197):

*“In the models, the drug clearance terms accounted for all types of drug metabolism which were not explicitly modelled (e.g. hepatic CYP450 activity) and i) renal elimination for the Blood compartment, ii) intestinal elimination for the Organs compartment and iii) biliary excretion for the Liver compartment only for irinotecan and 5-fluorouracil since it could be neglected for oxaliplatin [28-30]”.*

**Figures:

1. Figure 4: PK model of CPT-11: Blood compartment for SN-38 shows no clearance from blood (no clearance arrow CSN,B from BloodSN38), but the equation in SI (Eq1_(4)) does indicate a clearance of SN-38 from blood compartment (…-Csnb *Bsn). SAs in the text Line 200, SN-38 is eliminated by UDP-glycosyltransferases. These discrepancies need to be corrected.**

Answer: The equations in the SI have now been changed to correctly represent the model used.

**2. Figure 5: PK model of OXI: There is an efflux arrow from Organ to Blood compartment for free OXI. However, no corresponding efflux from Organ to Blood for free OXI is shown in Equation 1.2 (9) in their SI. The authors either need to correct the model scheme or equation to make sure it is consistent.**

Answer: The equations in the SI have now been corrected.

**3. Figure 6: There is no organ clearance in the scheme of their 5-FU model, which is consistent with what they explained in the text (Line 193) that 5-FU is mainly eliminated by liver through metabolism. However, the equation in SI 1.3 (15) shows organ clearance, so as their parameter estimate table SI table 8. This discrepancy needs to be corrected.**

Answer: These have now all been changed to be consistent with the model used.

**4. SI Figure 5 (b) and (d) show large range for that parameter estimate. How did the authors justify values in those cases?**

Answer: The parameter ranges shown on the identifiability plots are chosen to show the points at which the parameters cross the threshold. All values range to about 2-3 fold larger than optimal value and half to a third smaller than the optimal value.


**Minor Concerns/Recommendations:

Authors need to make sure that the symbols/abbreviations of their model parameters are consistent across text, equations, schemes, and tables!**

**We would like to thank the reviewer for their thorough review of our work and we have corrected all minor errors that they have brought to light.

Text
1. Line 124. The time delay for 5-FU is 2 h 202 min. Is it a typo of 202 min?**

Answer: This was a typo and has now been corrected. Line 147
**2. Line 186. It is the first time that SN-38 was written in the manuscript, however, the explanation of SN-38 shows later at Line 195.**

Answer: We thank the reviewer for pointing this out and a line has been added to explain SN38 in this sentence. Line 185
**3. Line 190. It is the first time that Pt was written in the manuscript, however, the explanation of Pt shows later at Line 202.**

Answer: This has been corrected. Line 261

**4. Line 164: Linear kinetics? Any references to support these assumptions?
Irinotecan follows linear PK, (conformed from the FDA drug label “Over the recommended dose range of 50 to 350 mg/m2, the AUC of irinotecan increases linearly with dose”) The linear assumption for irinotecan is true, but authors need to either justify in text or cite references properly. Apply to the other two drugs too, their linearity needed to be justified or properly cited.**

Answer: This was a misunderstanding in our use of the term “linear kinetics”. What we were trying to portray was that the mathematical terms in the equations were linear. We have now rephrased this so it is clearer (line 189-190):

*“All transports in between compartments were considered as passive and were represented by linear terms.”*

Next, we have now confirmed our CPT11 PK model does match the linear increase in AUC described by the FDA drug label which supports the validity of our model. This has been added in the SI and into the text (line 283-286): *“The irinotecan model had an almost perfect fit, it matched the linear increase of AUC compared to dose as described in the FDA drug label (SI Fig. 2) [39], and showed a rapid accumulation of both irinotecan and SN38 in the plasma of patients (Fig 6). ”*

This information is not available for 5-FU and oxaliplatin so the linearity has not been evaluated for them.

**5. Are the doses they used clinically relevant?**

**Yes, they are clinically relevant dosing regimen (confirmed by FDA drug labels of irinotecan and oxaliplatin and the reference of “Metzger G, et al. Spontaneous or imposed circadian changes in plasma concentrations of 5-fluorouracil co-administered with folinic acid and oxaliplatin: relationship with mucosal toxicity in patients with cancer. Clin Pharmacol Ther. 1994;56(2):190–201.”) However, authors need to properly justify the rationale of the doses they’ve chosen and cite the primary references.**

Answer: The drug doses and treatment schedules were designed as part of the OPTILIV clinical trial which integrated all available literature information and was approved by several regulatory bodies. Primary references have already been cited in the text (see references 20 and 37). We believe this is beyond the scope of this study to further elaborate on the clinical trial design.

**6. Line 403-414: Analytical method and assay validation were not explained or properly cited from the OPTILIV Study.**

Answer: Specific methods used for drug detection have been added to the text together with links to references with full method description: (line 480-493) *“Plasma pharmacokinetics (PK) data was gathered after the first dose of irinotecan, oxaliplatin and 5-fluorouracil and measured longitudinally for each individual patient. Plasma concentrations of irinotecan and its active metabolite SN38 were determined, using high performance liquid chromotagraphy (HPLC), in mg/ml at the start of infusion, then at 2, 3, 4, 6, 8 h 15 min and 31 h 45 min post HAI onset, for a total of seven time points, including baseline. Oxaliplatin concentrations were determined by measuring platinum plasma levels using spectrophotometry, for both unbound and total platinum levels. Oxaliplatin binds to proteins in the blood and the free Pt fraction is the biologically active one. Thus, oxaliplatin concentrations were determined at the start time of infusion, then at 3, 6, 9 h, 11 h 30 min and 17 h 15 min post HAI onset, for a total of six time points, including baseline. Plasma concentrations of 5-fluorouracil490were determined using HPLC, at the start of infusion, then at approximately 3 h, 5 h 45min, 9 h and 11 h 30 min post HAI, for a total of five time points, including baseline. The exact method used to assess plasma concentrations can be seen in Levi et al paper of the OPTILIV study [20].”*

**7. Line 198: Although they use MM PK, but the highest detected SN38 concentration in the OPTILIV paper are much smaller than its km (supported by reference No.28). Therefore, it is an apparent first order transfer from CPT-11 to SN38. It is okay to still use MM equation, but instead if they use linear PK, it could give them a simpler model and an acceptable way to reduce their over-parameterization problem**.

Answer: The Km value of CPT11 conversion into SN38 should be compared to the concentration range of CPT11 which is the substrate of the reaction and not to that of SN38. CPT11 concentrations are in the range of 1-2 µM and the Km is equal to 52 µM which is low but within an applicable range for Michaelis-Menten kinetics.

**Figures
1. The numbers of all figures were not correctly cited in the text. For example, page 5, Figure 1 (a) shows OXI concentration profile in the infusion tube. However, it should be Figure 2. This also applies to other figures**.

Answer: Figure 1 was uploaded as Figure 10 during online submission which explains the wrong figure numbers. We apologize for this and have now rectified figure indexes.

**2. Figure 2 (supposed to be Figure 3 in text): What about the (volume) flow rate? With only information of amount rate, there is no way to differentiate flow rate and drug dose, please also include flow rate figures. More general, there should be table for each of the three drugs with: total dose, drug concentration in infusion solution, total volume infused, total infusion time as well as peak and average flow [mL/min] and dose rate [mg/hr]. Considering the tubing dead space (1.85 mL, as mentioned on page 5), this information may better put in perspective/explain, why the delay is worst for OXI and least for CPT-11 - as shown on this and the subsequent figure.**

Answer: The original flow rates can be seen in Figure 1, panel A already. A table has now been added in the main text showing, for each drug, the total dose, drug solution concentration, and peak flow rates of main and spike deliveries (page 6).

**3. Figure 2 C, E G, the intended/nominal and simulated profiles have been mislabeled. Otherwise, it is not indicating lag-time, but action before drug on board. If so, what does that mean then?**

Answer: This has now been corrected.

**Equations
1. What does “d(t)” represent? Presumably the input function in amounts/time?**

Answer: *d(t)* is the rate of drug infusion as defined in the main text (line 132). This has now been explained also in the SI:

*“d(t) refers to the drug infusion function equal to the explicit solution of the PDE-based pump-to-patient model”*
**2. Equations 1 and 7 are incomplete with missing open parentheses.**

Answer: The open parentheses have now been added to equation 1 and 7, see SI.

Response to Reviewer #3’s comments

**Specific comments:

1. It might be useful to describe in the introduction why data from the OPTILIV study was chosen as opposed to other studies which are better parameterized. Does this study reflect commonly available information which might lead to better generalizability of the approach?**

Answer: A sentence has now been added to make the motivations behind using the OPTILIV dataset clearer, line 72-74:

“*This uncommon delivery route into the hepatic artery and the use of an infusion pump to deliver the drugs according to chronomodulated profiles represent a novel chemotherapeutic approach which needs to be quantitatively investigated to maximize patient benefit*.”

**2. The introduction describes significant sexual dimorphism with regards to specificity in previous trials – is this something that might be addressed in the PK modelling? Was gender a factor in the clustering of inter-individual PK differences? It would seem that this is an important factor that must be considered in some way, and merits some discussion if not explicitly examined in this study.**

Answer: First, gender was used as part of the scaling method to estimate patient-specific compartment volumes in the PK models (see line 185-187). Next, gender was considered as one of the possible covariates which could be linked with the patient clustering based on PK parameter values. However, no significant correlation was found which may be due to the small number of patients considered in this study. This has now been stated in a clearer fashion in the text, Line 334-335 “Clustering was compared to covariates of patients, such as gender, age and gene polymorphism, to see if there was any correlation however none was found.” and line 433-435:

*“In order to do so, patient clusters were compared to known covariates such as age, gender and gene polymorphisms. However, none showed significant correlation.”*

**3. In the introduction, the authors state that “Chronotherapy requires the error in drug infusion timing not to be greater than few minutes” – what is the evidence for this? Metabolic studies suggest that oscillations are the order of hours, so how and why must this so called chronotherapeutic window be narrowed so strictly?**

Answer: We agree with Reviewer #3 that “few minutes” is an overstatement and have now reworded this as “few hours”. Line 61-62.

*“Chronotherapy requires the error in drug infusion timing not to be greater than few hours.”*

**4. Does the use of glucose in the final flush have an impact on any of the key parameters which have been determined as glucose will have significant impacts on metabolism.**

Answer: The glucose flush involved a 5% glucose solution so that the entire flush corresponded to an administration of approximately 0.6g only. This is a non-significant amount for any metabolic reaction to be elicited.

**5. With regards to the PK modelling, it is noted that the effect of circadian rhythms was neglected to avoid over-parameterization. While this is reasonable, it is also somewhat inconsistent with the earlier statement on the top of page 3 that ADME profiles can have several folds difference within a 24 cycle. Thus the limitations of not including the circadian profile must be clearly stated.**

Answer: This limitation has now been added to the discussion as requested, line 413-415:
“*Furthermore, the current models do not account for any circadian rhythms although they may largely impact on drug PK-PD. Thus, new circadian clinical studies are needed to improve the models towards drug chrono-administration optimization.”*

**Minor comments:

‘Figure 1’ on page 5 appears to correspond to the actual Figure 2—not clear what the actual Fig 1 represents, particularly Fig 1A. In fact, all figures are misnumbered by 1, with the actual Fig 1 not referenced in the text.**

Answer: This was an error in the uploading process and we apologise for any confusion. Figure 1 in your PDF should actually have been Figure 10. All figures are now referenced correctly.

**Fonts on real Figure 4 are distorted (e.g. efflux reads as eff ux etc.).**
Answer: This has now been rectified.

**X-axis on real Figure 10 is upside down**

Answer: The number of ticks of the x-axis has now been reduced so the numbers can be presented horizontally to prevent the axis from looking upside down.

**Line 335 vhronotherapy --> chronotherapy**

Answer: This has now been corrected (line 406).
