# Supplementary material for: Optimizing circadian drug infusion schedules towards personalized cancer chronotherapy
Source: PLoS Comput Biol. 2020 Jan 27;16(1):e1007218. doi: 10.1371/journal.pcbi.1007218 (PMC7004559; doi:10.1371/journal.pcbi.1007218)
Supplement: Attachment — Submitted filename: Response_to_reviewers.odt [file pcbi.1007218.s003.odt]

Reviewer #1: Most of the reviewers' concerns have been addressed.
Some minor issues/errors still need to be curated before being published:
	Response: We thank the reviewer for bringing these final errors to our attention. We have now corrected the errors listed below.
1. The "wij" of the formula between line 575-576 should be "wij^2". Since the formula between line 569-570 in this article is equivalent to the formula (7) in Ref [40], the m=2 is used in this article; the formula between line 573-574 in this article is equivalent to the formula (5) or (10) in Ref [40], which further shows that the wij in this article is actually the uij in Ref [40]. However, the formula (17) in Ref [40] shows the uij has an exponent m, but the formula between line 575-576 in this article shows the wij has no exponent m. If m=2 is used, "wij" should be "wij^2".
	Response: The reviewer is correct this should have been "wij^2". This was a typo and has now been corrected (line 575-576).
2. The description in line 311-314 is not correct. It should be "In all three models the parameters which showed the largest inter-patient variability were transport parameters specifically, for irinotecan Blood-Organ transport (CV = 176.3), for oxaliplatin Blood-Liver transport (CV = 318.2) and Blood-Liver/Organ transport for 5-fluorouracil (CV = 187.52)."
	Response: We thank the reviewer for noticing this error we have now corrected it sentence to read “In all three models the parameters which showed the largest inter-patient variability were transport parameters specifically, for irinotecan Blood-Organ transport, for oxaliplatin Blood-Liver Transport and Blood-Liver/Organ transport for 5-fluorouracil.” (line 311-314).
3. Figure 5 still shows "eff ux" instead of "efflux"
	Response: This has now been changed to K_b,l/o in align with the other diagrams.
4. Ref [21] was not the only hyperlink which doesn't work. Although it is fixed, the hyperlinks in Ref [39] and [44] still don't work.
	Response: We thank the reviewer for noticing this issue, this is a problem with our reference organising software and has now been corrected.
